# Fuzzy Logic and Genetic-Based Algorithm for a Servo Control System

**DOI:** 10.3390/mi13040586

**Published:** 2022-04-09

**Authors:** Hugo Torres-Salinas, Juvenal Rodríguez-Reséndiz, Edson E. Cruz-Miguel, L. A. Ángeles-Hurtado

**Affiliations:** 1 Facultad de Informática, Universidad Autonóma de Querétaro, Querétaro 76230, Mexico; htorres@ieee.org; 2 Facultad de Ingeniería, Universidad Autónoma de Querétaro, Querétaro 76010, Mexico; langeles11@alumnos.uaq.mx; 3 Facultad de Ingeniería en Electrónica y Comunicaciones, Universidad Veracruzana, Veracruz 91090, Mexico; edsoncruz@uv.mx

**Keywords:** position controller, artificial intelligence, intelligent control, fuzzy controller, genetic algorithm, optimized controller

## Abstract

Performing control is necessary for processes where a variable needs to be regulated. Even though conventional techniques are widely preferred for their implementation, they present limitations in systems in which the parameters vary over time, which is why methods that use artificial intelligence algorithms have been developed to improve the results given by the controller. This work focuses on implementing a position controller based on fuzzy logic in a real platform that consists of the base of a 3D printer, the direct current motor that modifies the position in this base, the power stage and the acquisition card. The contribution of this work is the use of genetic algorithms to optimize the values of the membership functions in the fuzzification of the input variables to the controller. Four scenarios were analyzed, in which the trajectory and the weight of the system were modified. The results obtained in the experimentation show that the rising and setting times of the proposed controller are better than those obtained by similar techniques that were previously developed in the literature. It was also verified that the proposed technique reached the desired values even when the initial conditions in the system changed.

## 1. Introduction

Position controllers are implemented in systems where it is required to reach a specific point or follow a predefined path within a workspace. The main objective of regulating the position is to carry out controlled displacements within the system, thereby reducing the difference between the desired value and the current value of the variable of interest. The many applications of these controllers include as conveyor belts in manufacturing systems, industrial welders, automatic product labeling and robotic systems.

PID controllers are preferred in the industry due to their robustness, simplicity of design and implementation [1,2,3,4,5]. Despite the above, these techniques have disadvantages, showing poor performance in systems where the mathematical model contains an integrator or an unstable transfer function. They are sensitive to external disturbances and resonances during the process [6]. Control strategies have been developed to solve the above-mentioned problems that combine conventional methods with artificial intelligence, such as fuzzy logic, genetic algorithms, and neural networks [7]. These hybrid technologies have proven to be effective, significantly improving the performance of the systems, reducing the time needed to reach the desired value, and reducing energy costs.

The motivation to carry out the investigation presented in this article lies in finding a solution that uses the previously mentioned techniques to reach the desired values in processes where the initial conditions are modified. The main objective is the design of a servo position controller and its implementation in a real experimentation platform consisting of a 3D printer. The problem is that the gains in traditional controllers remain fixed even when the conditions of the process for which they were tuned change. Therefore, the main contribution proposed in this work is a technique by which the behavior of fuzzy controllers is improved using artificial intelligence, specifically, a GA, to optimize them. Each individual in the GA is composed of the parameters of the membership functions of the fuzzy controller, seeking the best values for them. Therefore, these values are modified if the conditions of the system are also modified. In addition, it is also possible to reduce human errors in the fuzzification process.

The test platform consists of a commercial 3D printer base, whose primary actuator was changed for a direct current motor. It is also necessary to add the data acquisition elements and the power stage. The experimentation is designed in four scenarios, in which the trajectory and the weight that the printer must move are modified, thus verifying the efficiency of the controller when the initial conditions are modified. The results showed that the technique proposed here manages to achieve the desired position values in all the scenarios in which it was tested. In addition, these results are compared with previous works in the literature that use similar techniques and it was found that the performance of the controller improves the rise and settling times. Even when previous works use the same approach, the presented proposal offers the following contributions:

- A fuzzy controller that uses a GA to optimize its membership functions.
- A platform for real experimentation that promotes knowledge in electronics and programming.
- Good control results in terms of rising and settling time and overshoot.
- The controller adapts to variations in the initial conditions.
- Better behavior compared to similar techniques previously used in the literature.

The rest of the paper is organized as follows. Section 2 is dedicated to reviewing works related to the proposal made in this research. Section 3 grants the theoretical framework necessary to understand the fuzzy logic and genetic algorithms used in the hybrid controller. The methodology is discussed in Section 4, where a complete description of the experimentation platform is provided. The methodology also describes the fuzzy controller design and how it is optimized by the genetic algorithm. Section 5 shows the results obtained by the proposed controller in the experimentation scenarios. Section 6 addresses the discussion, the takeaways, and the implications of the results obtained in this research. Finally, Section 7 discusses the conclusions and possible future work that improve the current investigation.

## 2. Related Works

This section mentions previous research related to control methods used to solve problems related to unstable systems, disturbances, and nonlinear behavior [8,9]. In the literature, different techniques have been developed to solve the problems of classic controllers using recent phenomena such as the IoT. These techniques have delivered outstanding results, since the IoT allows different sensors and actuators to be connected, achieving the design of more complete systems [10,11]. Novel proposals have been presented that seek to actively eliminate external disturbances in the system through nonfragile controllers, which consider several known problems within their design, such as the uncertainty of the model parameters, faults in the system elements and measurement errors. These nonfragile controllers have overcome each of the above hurdles [12,13].

Recent works have shown that fuzzy logic improves the performance of conventional controllers in the presence of disturbances and nonlinear behavior [14,15,16]. It has been found that the action of actuators can be reduced by up to 50% by running a fuzzy controller instead of a PID controller, thereby reducing energy consumption and associated costs [17,18]. Researchers have used fuzzy logic to identify system parameters and modify controller gains because its implementation is relatively simple. Unlike strategies such as sliding mode control, fuzzy controllers do not require a complex mathematical model of the plant [19]. Therefore, fuzzy logic has become one of the preferred techniques for use in processes in which control is required [20,21]. However, it has several limitations, such as the difficulty of interpreting fuzzy values. It requires multiple rules and, finally, a person with a high degree of knowledge about the behavior of the system. The last-mentioned limitation is significant since the possibility of human errors in the controller design process is high.

Hybrid controllers have been developed to overcome the limitations of fuzzy logic. These controllers add additional algorithms that have shown an effective and improved system performance on multiple targets [22]. Artificial intelligence has played a fundamental role in creating hybrid techniques, including ANN, ACO, SVR, and PSO algorithms. These algorithms have significantly improved in terms of aspects such as smoothing the trajectories of the actuators, reducing energy consumption, and achieving shorter operating times [23,24,25,26,27].

Proposals have previously been presented, in which a hybrid controller with fuzzy logic and GAs was designed. They achieve the desired objectives in the systems and improve their performance against nonlinear behaviors [5,28,29,30]. These techniques have also proven to be effective in counteracting disturbances and changes in the initial conditions of the process. These achievements allow the controller to modify its profits if there are changes in the original process [31,32,33]. As can be seen, the previous methods obtained excellent results and are very similar to the one proposed in this article. However, the main motivation that inspired this research is the desire to design a hybrid controller and implement it in a real experimentation platform, unlike previous works that test its performance through simulation. In addition, our proposal provides competitive rising and settling times compared to other techniques.

## 3. Theoretical Framework

This work uses the union of fuzzy logic and genetic algorithms to design a hybrid controller that regulates the position. Therefore, this section aims to briefly describe the fundamentals necessary to understand both techniques.

### 3.1. Fuzzy Logic

The father of fuzzy logic, Lofti A. Zadeh, presented the work “fuzzy sets” in 1965, in which the belonging of objects were defined through classes [34]. The fuzzy sets allowed for the classification of persons into sets such as “the class of short men” or “the class of young women”, and, for this reason, Zadeh considered the sets he defined to be closer to human reasoning, mainly regarding issues of communicating or abstracting information and pattern recognition. Since then, a wide variety of works have been developed in different research areas and have demonstrated the strengths of this technique, such as:

- Fuzzy sets are relatively easy to implement.
- A complex mathematical model of the process is not necessary.
- They have a low computational cost.
- They can be implemented to develop MIMO systems.

Fuzzy logic is divided into three phases: the fuzzification, the inference and the defuzzification [35].

#### 3.1.1. Fuzzification

The fuzzification stage is responsible for transforming numerical variables into linguistic variables. For this process, each variable has a degree of membership in the range of 0 (does not belong) and 1 (totally belongs). This degree of membership is defined in Equation (1), where *A* is a fuzzy set and *x* is a value of the universal set *X*.

(1) $$\mu_{A}\left(x\right):X\to \left[0,1\right]$$

To understand Equation (1), it is necessary to define the following terms:

- *X* is the universe of discourse and represents the domain of the variable *x*, that is, each of the values that it can take.
- A fuzzy set is a set in which an element *x* can partially belong to set *X*. In this way, an element can belong to several independent fuzzy sets.
- A crisp set is a conventional set, it is the binary type, and an element can only be inside or outside it.
- The membership functions are the sets to which a variable has a greater or lesser membership. These sets have varied shapes but are mainly triangular, trapezoidal, singleton, and type S.

#### 3.1.2. Fuzzy Inference

Fuzzy logic uses rules for decision-making. These rules are made up of fuzzy inputs, also known as precedents, and outputs, also called consequences. Precedents and consequences are associated with if-then statements [36].

Fuzzy rules are generally grouped into tables, although representation can be difficult when the input variables are more than two. The different input variables are associated using the logical operators AND, OR, and NOT.

The two main groups of fuzzy rules are the Takagi–Sugeno rules and the Mandani rules. Each of these groups has different advantages, and the main difference between them is that the output of fuzzy inference in the Mandani method is still a fuzzified variable, while in the Takagi–Sugeno rules, the output variable is already a crisp value [37].

#### 3.1.3. Defuzzification

A common and widely used method is the centroid, in which the fuzzy output is transformed into a real number that represents the center of gravity of such a fuzzy output set [38]. However, more efficient methods, such as the COA, reduce the computational load required to carry out this process.

### 3.2. Genetic Algorithms

A genetic algorithm is a method that is mainly used to solve optimization problems. They are inspired by the biological behavior of living things, specifically their reproduction. The goal of GAs is to mimic biological evolution to obtain better solutions in better times. In general, GAs are part of the so-called artificial intelligence: solving problems through computer programs that mimic the functioning of natural intelligence [39].

The GAs are presented as a global search optimization technique. They explore a great variety of possible solutions for the problem they are trying to solve, and, therefore, manage to avoid optimal local solutions to go in search of optimal global solutions [40]. The following terms describe basic concepts for optimization problems.

- The objective function is a function that seeks to minimize or maximize its value and can also contain multiple variables.
- An optimal local solution is defined as the maximum or minimum only for a delimited region of all the available solution space.
- The optimal global solution represents the maximum or minimum for the entire solution space of the objective function.

GAs work with a population of individuals or a set of possible solutions to the problem. This set is subjected to random actions, similar to those that occur in biological evolution, to improve the population. Genetic mutations or re-combinations are among these actions. Evolution also occurs when a selection is made according to a criterion establishing which are the fittest individuals, which will survive, and which are the least fit, which is discarded [39].

#### 3.2.1. Initialize Population

An initial population that contains possible solutions to the problem is randomly generated. These solutions are called individuals. Each of the individuals must be coded on a chromosome that contains several genes that correspond to each of the parameters of the problem. In order for a computer to understand chromosomes, they must be encoded in a chain; that is, a series of numbers, letters, or a combination of both.

The correct choice of encoding is one of the critical points to obtaining an excellent solution to the problem under study. Therefore, there are several ways to carry out this process, such as binary encoding, finite value encoding, or the use of integers [41].

#### 3.2.2. Evaluate the Objective Function

Each of the individuals generated above is evaluated by the objective function, usually a mathematical equation. This objective function gives a score to the individuals who provide the best solutions. Although most of these solutions do not work, a few may be promising. They may show part of the solution, even if it is weak and imperfect [42].

#### 3.2.3. Selection

Once the individuals have been tested, the GA must decide which ones should be chosen to breed. There are different techniques to carry out this process. These techniques use a combination of the probability and aptitude of each individual [43]. The main selection techniques are:

- Elitist selection: the selection of the best individuals of each generation is sought.
- Selection proportional to aptitude: the best individuals are more likely to be selected. However, there is also a probability that less suitable individuals are chosen.
- Roulette wheel selection: the probability that an individual is selected is proportional to the difference between its fitness and the rest of the individuals.
- Selection by tournament: individuals in the population compete directly with each other, and only one is chosen from each competition.

#### 3.2.4. Reproduction

In reproduction, the algorithm seeks to follow the example of nature, where genetic diversity is present through sexual reproduction. In GA, a pair of individuals cross their genes and generate two children that combine the characteristics of both parents. Crossing, or reproduction, is synonymous with mating between two individuals of a different sex [42].

Techniques such as those described below can be used to perform the reproduction:

- Crossing of a point: a point of exchange is randomly defined in the chromosomes of the two individuals. The first individual contributes all his previous genes to that point, and the other individual contributes her genes from that point on.
- Crossover at two points: This technique is similar to the previous one, but now, individuals exchange genes at intervals delimited by two points.

#### 3.2.5. Stopping Criteria

Ideally, the AG should stop when the optimal solution is reached, but this solution is usually unknown. Therefore, several stopping criteria are used: that the objective function reaches a previously defined acceptable value, that the algorithm meets a certain number of iterations, or that it reaches a specific operating time.

## 4. Methodology

**Figure 1:**
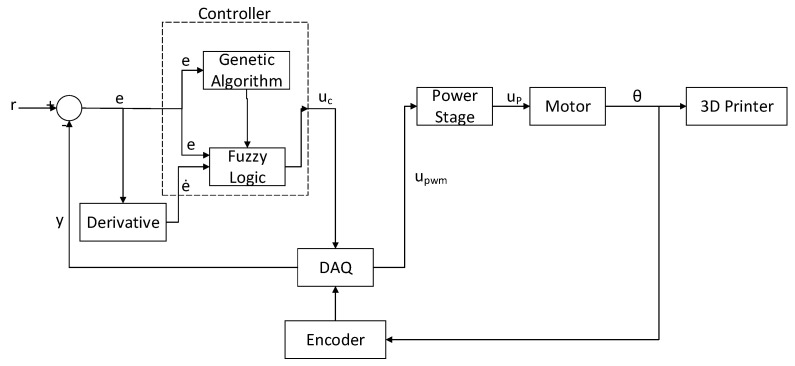
Diagram of the control loop designed for the project.

As previously mentioned, the objective of this work is to control the position of a 3D printer. The methodology focuses on developing each block shown in Figure 1, which shows the diagram of the control loop designed to achieve the last objective.

**Figure A1:**
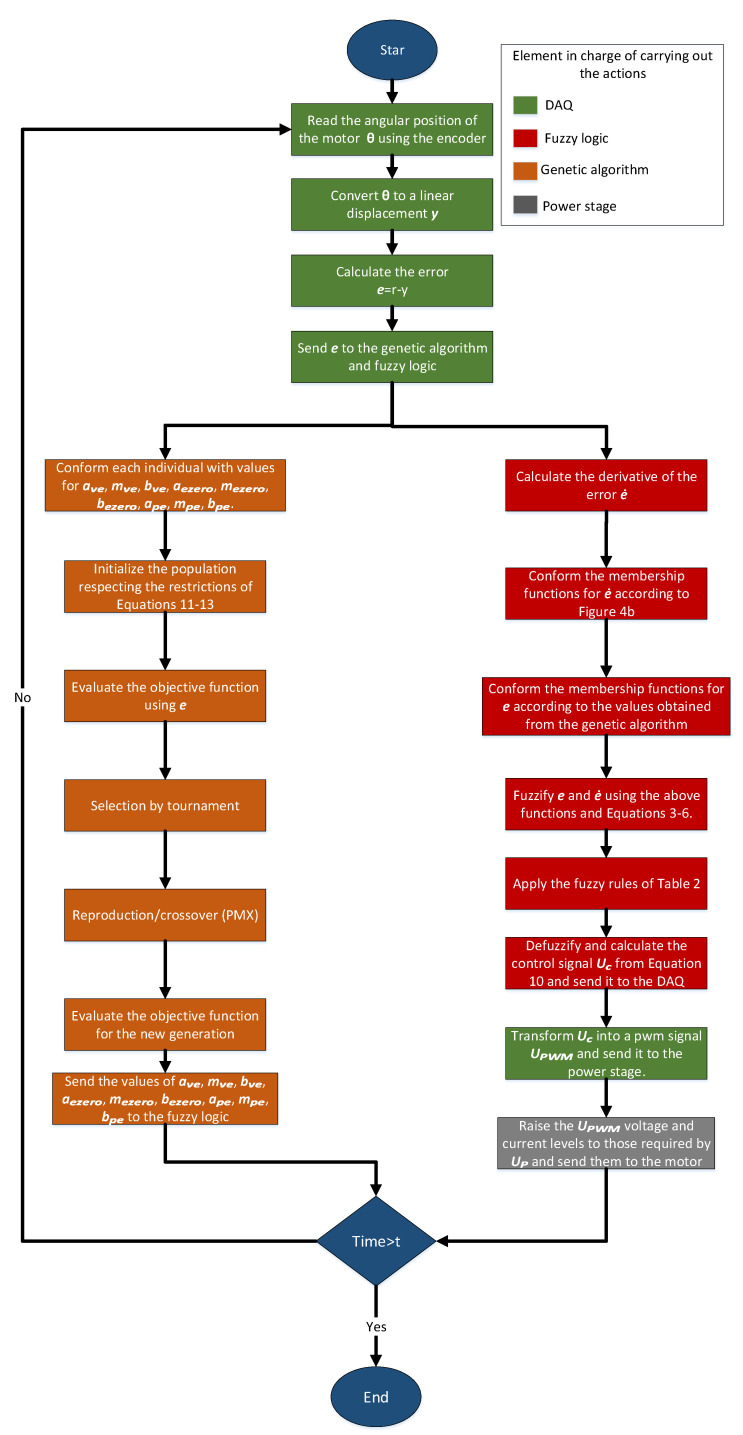
Flowchart designed for the project.

The controller block comprises a hybrid technique between fuzzy logic and genetic algorithms. The DAQ is in charge of converting the signals to adequate values so that the computer can understand the measurements. The power stage raises the voltage and current levels delivered by the DAQ, and thus makes the system capable of moving the selected motor. This motor, in turn, is the actuator that modifies the position of the base in the printer. Table A1 shows the variables used in Figure 1. Figure A1 shows how the mentioned variables interact with each other, as well as the workflow of the methodology.

### 4.1. Description of the Study Platform

**Figure 2:**
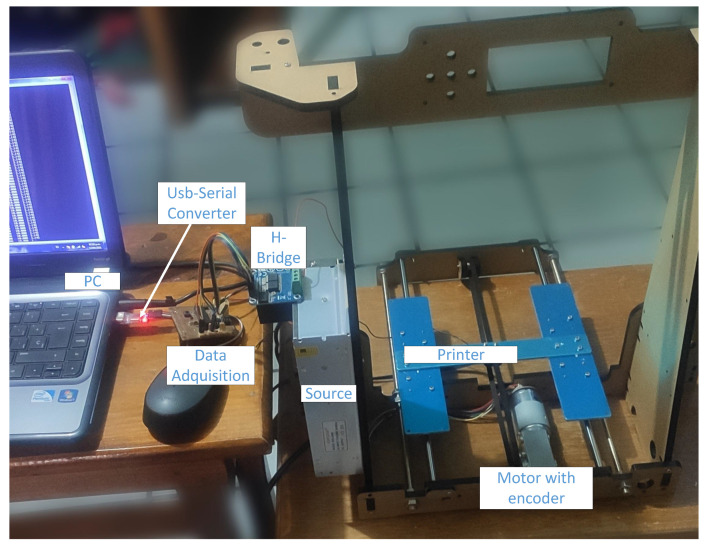
Experimentation platform designed for the project.

The objective of this section is to describe the experimentation platform designed for the project. This platform is an extensive model of the work presented in [44]. Both experimentation platforms share several of the devices used for the operation. Figure 2 shows a real representation of the elements that make up the platform. The function of each element is briefly described below.

The platform actuator is a direct current electric motor. Electric motors are widely used in multiple servo control applications. In this case, the actuator works in conjunction with a rotary encoder to transform the angular movements θ into pulses that are proportional to the revolutions in the motor shaft.

To satisfy the needs of the project, it is necessary to use an element that increases the levels of the control voltage $u_{pwm}$ to those required by the motor. This required voltage is represented by $u_{P}$. An H-bridge is used to achieve this function.

In this investigation, the base of a 3D printer model Anet A8 is used to test the performance of the controller. The motor has a toothed coupling added to its shaft. This coupling has the function of holding an elastic band that is directly connected to the printer base. Each motor shaft rotation corresponds to a linear movement *y* in the platform. The relationship that describes this movement is shown in Equation (2).

(2) 1revolution=360=1.5cm=256PPR

The variable *y* has a complete range of 30 cm, which is deliberately restricted to leave a 3 cm safety margin on each side.

The acquisition card has several fundamental functions within the platform. It is the element in charge of linking all the components that are necessary for the operation of the project. Due to its high processing speed, a DSC was selected as the main device in the DAQ. The sampling time $T_{s}$ is 5 milliseconds. The tasks of the card are the following:

1. Reads the position of the motor through the encoder coupled to its shaft.
2. Converts the pulses delivered by the encoder to a linear displacement *y*.
3. Sends the position to the controller.
4. Receives the control signal.
5. Converts the control signal to PWM and sends it to the H-bridge. This signal is labeled as $u_{pwm}$ in Figure 1.

### 4.2. Controller

This section details how the controller is designed. The fuzzy part and the optimization by genetic algorithms are explained in detail and require an understanding of Section 3 of this document.

#### 4.2.1. Fuzzy Controller

Membership functions of the triangular, trapezoidal, L-type, and R-type were used to transform the numerical values of the variables to linguistic expressions. The equations used to find the degree of membership are shown in (3)–(6).

- Equation (3) corresponds to the triangular function.

(3) $$\mu_{A}\left(x\right)=\left\{\begin{array}{ll} 0, & \mathrm{if}\;(x\leq a)\\ \frac{x-a}{m-a}, & \mathrm{if}\;(a<x\leq m)\\ \frac{b-x}{b-m}, & \mathrm{if}\;(m<x<b)\\ 0, & \mathrm{if}\;(x\geq a) \end{array}\right.$$

- Equation (4) defines the trapezoidal function.

(4) $$\mu_{A}\left(x\right)=\left\{\begin{array}{ll} 0, & \mathrm{if}\;(x<a)\;or\;(x>d)\\ \frac{x-a}{b-a}, & \mathrm{if}\;(a\leq x\leq b)\\ 1, & \mathrm{if}\;(b\leq x\leq c)\\ \frac{d-x}{d-c}, & \mathrm{if}\;(c\leq x\leq d) \end{array}\right.$$

- The R-Function is described in Equation (5).

(5) $$\mu_{A}\left(x\right)=\left\{\begin{array}{ll} 0, & \mathrm{if}\;(x>d)\\ \frac{x-a}{b-a}, & \mathrm{if}\;(a\leq x\leq b)\\ 1, & \mathrm{if}\;(x<c) \end{array}\right.$$

- Equation (6) corresponds to the L-Function.

(6) $$\mu_{A}\left(x\right)=\left\{\begin{array}{ll} 0, & \mathrm{if}\;(x<a)\\ \frac{x-a}{b-a}, & \mathrm{if}\;(a\leq x\leq b)\\ 1, & \mathrm{if}\;(x>b) \end{array}\right.$$

Figure 1 shows that the error and the derivative of the error enter the fuzzy part of the controller. The error is obtained utilizing the difference between the desired value *r* and the real value *y*, as shown in Equation (7).

(7) e=y−r

**Figure 3:**
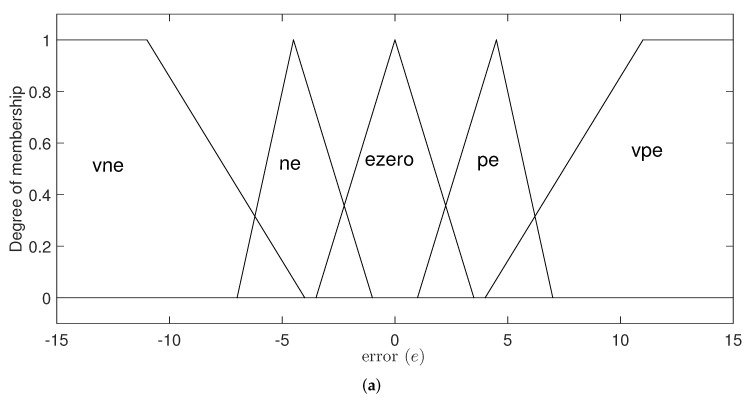

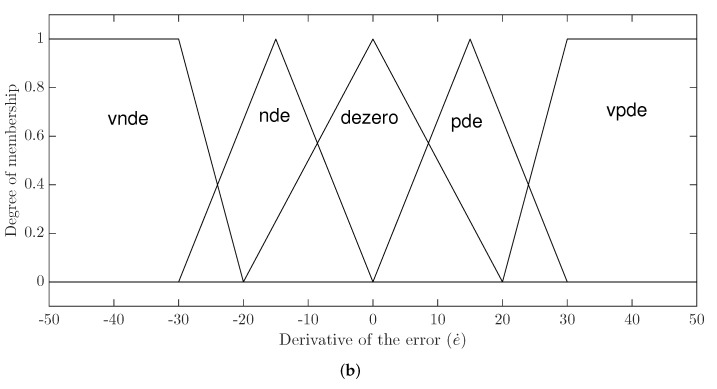
Membership functions for input variables. (**a**) Error; (**b**) derived of the error.

Three triangular functions are used for the error variable (*e*), an R-type function, and an L-type function. The universe of discourse has a range from −15 to 15 cm. The distribution for this variable is shown in Figure 3a. It is important to emphasize that the values are not fixed for the triangular functions but instead connected with the genetic algorithm and are updated depending on the factors explained later in this document.

The second input variable is the derivative of the error($\dot{e}$). For its fuzzification process, five membership functions are used: an L-type function, an R-type function, and three triangular functions. The universe of speech ranges from −50 to 50 cm per second. This distribution is shown in Figure 3b.

**Figure 4:**
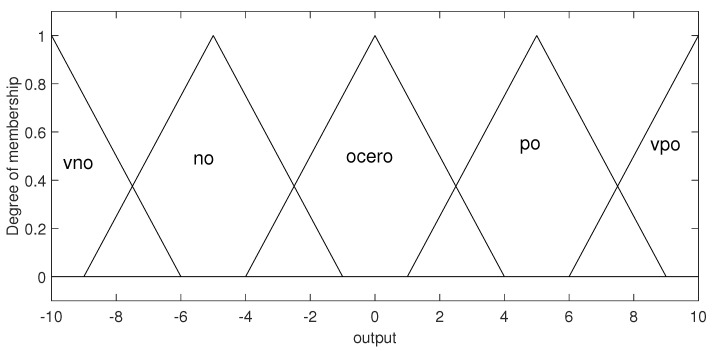
Membership functions for the output.

There is a single output variable, and five membership functions of the triangular type are used to transform its numerical value into linguistic expression. The triangular functions have a universe of discourse with a range from −10 to 10 V. Figure 4 shows the distributions of these membership functions.

The labels and their corresponding linguistic expression used for the membership functions are shown in Table 1.

The fuzzy rules were defined based on previous knowledge about the behavior of the system, taking a previous investigation carried out by the authors as a reference [44]. This knowledge was obtained through multiple experiments on the test platform, in which different types of controllers were implemented. As a result of the above, 25 rules were established, which are shown in Table 2.

The logical AND connector corresponding to the intersection operation described in Equation (8) is used to link the two input variables in the fuzzy rules where *T* is the triangular norm, also known as T-norm.

(8) $$\mu_{A\cap B}\left(x\right)=T\left(\mu_{A}\left(x\right),\mu_{B}\left(x\right)\right)$$

Since the T-norm used in fuzzy sets is the minimum value, Equation (8) is rewritten, resulting in Equation (9).

(9) $$\mu_{A\cap B}\left(x\right)=min\left(\mu_{A}\left(x\right),\mu_{B}\left(x\right)\right)$$

CoA is used in the defuzzification process. With this technique, the area of the membership functions, in which the fuzzy output obtained membership, is calculated according to Equation (10). This is the final stage of the controller and its output is $u_{c}$, where *x* is the value of the linguistic variable and its domain is represented by $x_{min}$ and $x_{max}$.

(10) $$u_{c}=\frac{\int_{x_{min}}^{x_{max}}f\left(x\right)xdx}{\int_{x_{min}}^{x_{max}}f\left(x\right)dx}$$

Simplified steps for the fuzzy controller are shown in Algorithm 1.
**Algorithm 1:** Pseudocode of the fuzzy controller.
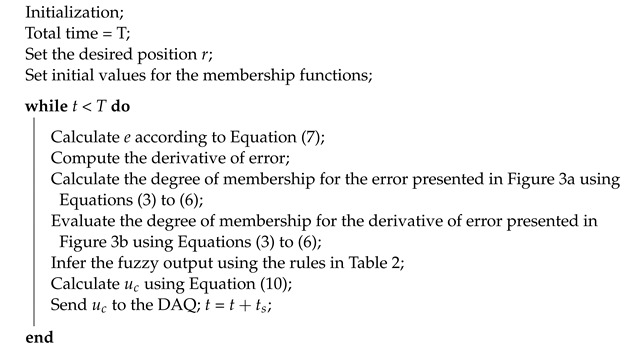


**Figure 5:**
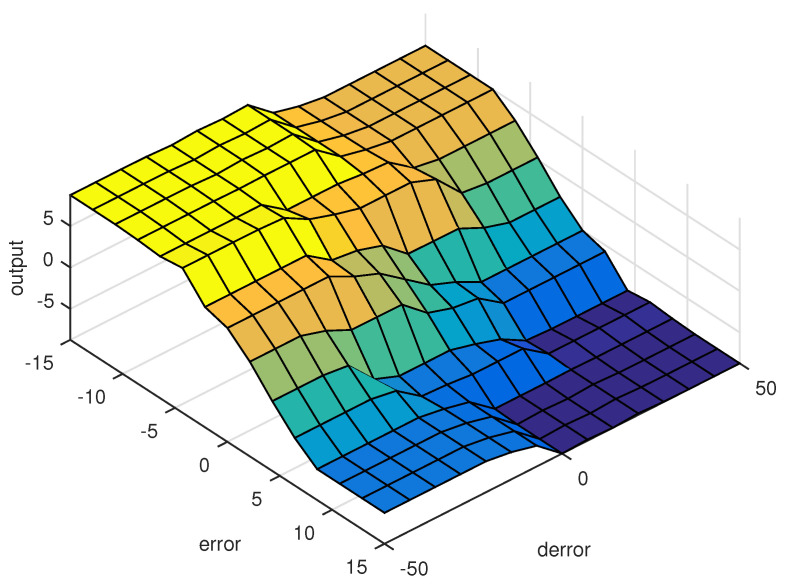
Proposed fuzzy control surface.

The control surface is helpful as it provides a quick overview of the general behavior of the designed controller. It is an essential tool for the selection of parameters. The graphical relationship between the inference rules and the fuzzy variables are shown in Figure 5.

#### 4.2.2. Optimization with Genetic Algorithms

As previously mentioned, the link between the fuzzy controller and the optimization with genetic algorithms is the input variable of the error. Specifically, the aim is to optimize the values for the triangular functions of Figure 3a that correspond to the linguistic expressions ne, ezero and pe.

**Figure 6:**
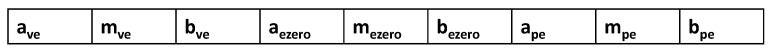
Representation of an individual for genetic algorithms.

Equation (3) describes three values that are necessary to represent a triangular function: the left base of the triangle represented by the letter *a*, the highest point of the triangle *m*, and finally the base of the triangle *b*. Figure 6 shows that each individual of the population in the genetic algorithms is made up of the combination of these three points for each of the three triangular functions that are going to be optimized.

For the initialization of the population, 40 individuals are randomly generated, sych as the one in Figure 6. To achieve a uniform distribution and maintain the triangular shape of the functions, the individuals must satisfy the conditions shown by Equations (11)–(13).

(11) $$-15\leq a_{ne}<m_{ne}<b_{ne}\leq 0$$

(12) $$-5\leq a_{ezero}<m_{ezero}<b_{ezero}\leq 5$$

(13) $$0\leq a_{pe}<m_{pe}<b_{pe}\leq 15$$

After generating the initial population, its aptitude is evaluated according to Equation (14) where r(k) and y(k) are the desired value and the value measured in iteration *k*, respectively, and *m* is the count of iterations.

(14) $$J=\frac{1}{m}\sum_{k=1}^{m}{\left[r\left(k\right)-y\left(k\right)\right]}^{2}$$

**Figure 7:**
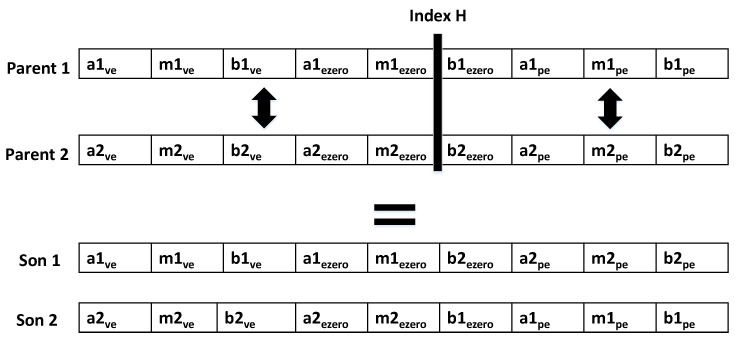
Demonstration of the PMX cross.

The next step is the selection process. For this step, the tournament method is used, which consists of randomly comparing two individuals from the initial population. The individual with the best aptitude is chosen to reproduce. Once the individuals have been selected the PMX cross is used, as shown in Figure 7. In this type of crossover, two individuals, known as parents, share genes based on an *H* index. The first new individual acquires all the genes of the first parent up to index *H* and, from this index, acquires the genes of parent 2. The second of the individuals acquires the genes of the second parent up to index *H* and the genes of the first parent after index. The above process is repeated until a new population is generated with the same number of individuals as the initial population.

Once a new generation has been created, its suitability must be re-evaluated. The process continues until the stopping criterion is met, which consists of completing 2 s of iterations for the experiments for a single desired value and 12 s for the experiments with multiple desired values.

The pseudocode for the genetic algorithm is shown in Algorithm 2.
**Algorithm 2:** Pseudocode of the GA.
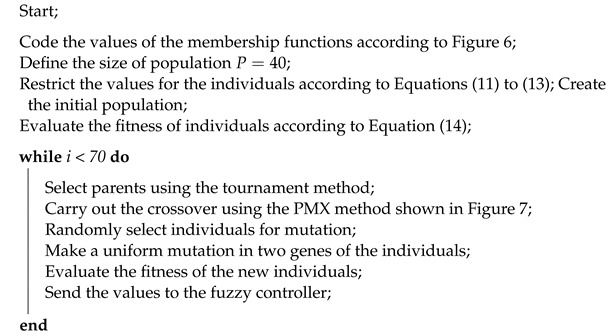


## 5. Results

The experimentation was designed to subject the controller to four different scenarios.

- **Scenario 1:** No weight on the base and define a trajectory for a single desired value.
- **Scenario 2:** No weight on the base and define a trajectory for several desired values.
- **Scenario 3:** Add 5-pound weight and define a trajectory for a single desired value.
- **Scenario 4:** Add 5-pound weight and define a trajectory for several desired value.

**Figure 8:**
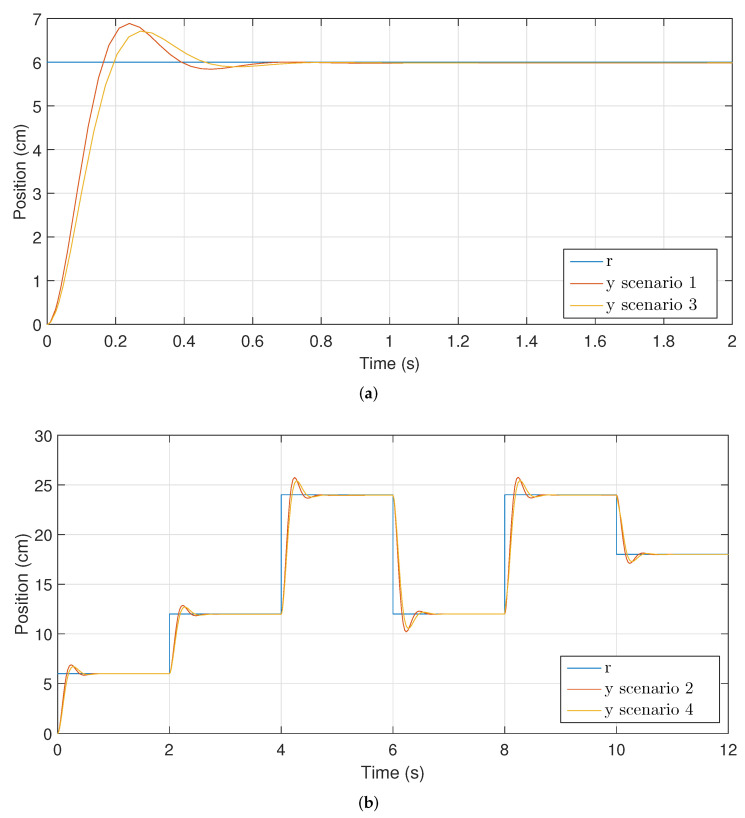
Controller behavior implemented in the experimentation platform. (**a**) Results for scenario 1 and 3; (**b**) results for scenario 2 and 4.

The results for scenario 1 and 3 are shown in Figure 8a and for scenario 2 and 4 they are shown in Figure 8b.

**Figure 9:**
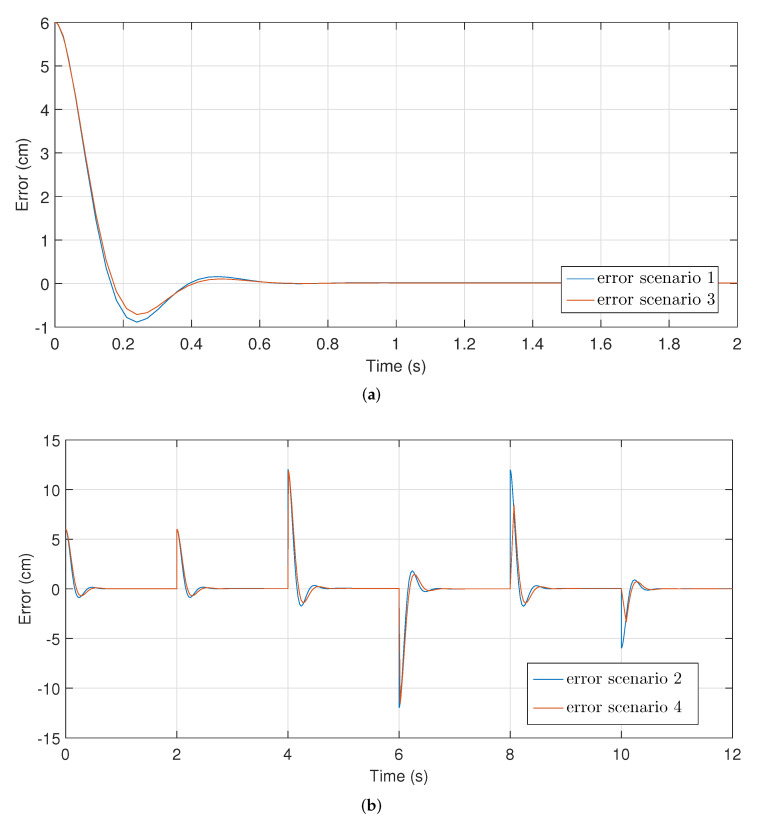
Errors obtained by the experimentation platform. (**a**) Errors for scenario 1 and 3; (**b**) errors for scenario 2 and 4.

**Figure 10:**
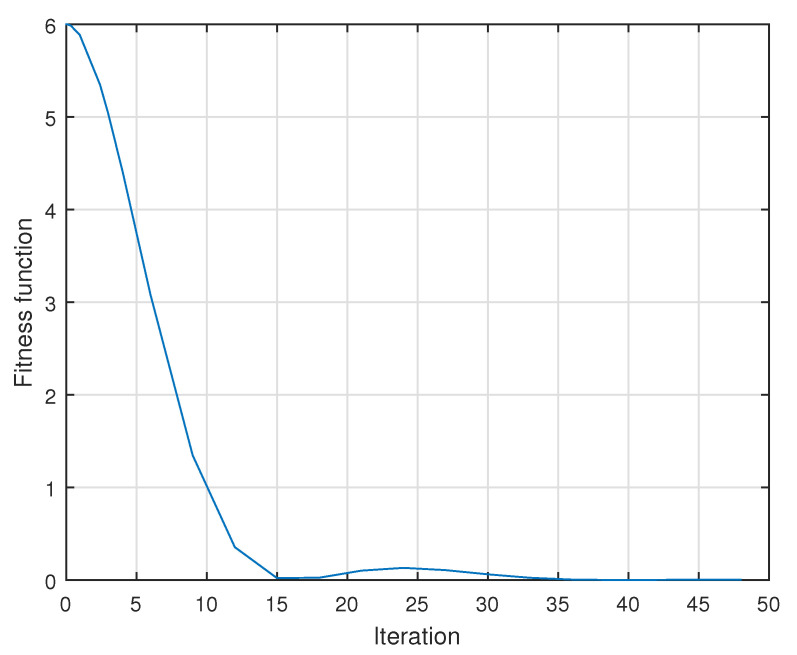
Reduction in the error in the iterations for the controller.

Figure 9 shows the estimated errors in each scenario and Figure 10 shows the reduction in the error in the system as the number of iterations increases.

## 6. Discussion

As shown in Figure 8a, the controller obtains the desired value and stabilizes, thus fulfilling the main objective in each of the experimentation scenarios. Figure 8b also shows that in scenario 1 the rise time is reached faster than in scenario 3. This difference is due to the weight added to the base of the 3D printer, which causes the system to move slightly slower. In both cases, the overshoot is less than 15% and the number of oscillations is small, implying that the energy consumption of the system is adequate.

Figure 9b displays that the controller manages to follow a predefined trajectory, reaching the reference value within the established time in each case. This result demonstrates the effectiveness of the controller in the face of variations in the initial conditions of the process.

Figure 9 shows the estimation of the errors obtained for each scenario, reducing these in each case. The oscillations presented in this Figure are analogous to those in Figure 8. All errors reach the desired value within less than 2 s. In the same way, the error is eliminated in cases where the objective is to follow a previously defined path. Finally, Figure 10 shows how the error decreases in each new iteration of the implemented algorithm.

The takeaways of the results can be synthesized as follows:

- It was possible to implement the controller in a real experimentation platform consisting of a 3D printer and data acquisition and power elements.
- The controller reaches the desired values even when the initial conditions of the system change.
- The error is reduced to 0 in each of the scenarios proposed.
- Rise and settling times are competitive compared to similar techniques in the literature.

Table 3 shows the results obtained in works that use similar techniques. The scenario with which it is compared is 1, since it has the most similarities with other papers in terms of the conditions under which the controller is subjected.

The relevance of the results implies that the proposal presented in this research is an excellent alternative to those previously designed since it promotes the formation of knowledge in AI, electronics, programming, and control. In addition, the results obtained show that the proposed technique effectively overcomes the limitations that motivated this research since even if the initial conditions of the system are modified, the controller still reaches the predefined references.

## 7. Conclusions

It can be concluded that the proposed hybrid controller is an excellent option to obtain the desired values. Although it showed slightly different behaviors, it can reach the desired value even when the initial conditions such as weight or trajectory are modified. Fuzzy logic has the limitation that once its parameters have been defined, it is impossible to modify them even if the dynamics of the system change. Adding artificial intelligence manages to overcome the previous limitation.

The proposed work develops the necessary knowledge to implement control methods on platforms that require skills in power and digital electronics, and micro-controller programming. The hybrid controller implemented in this paper offers a novel technique by optimizing, through genetic algorithms, the membership functions used in the fuzzification of the system. In addition, the results obtained showed that the rise and settling times are better than those obtained by similar techniques previously shown in the literature. Due to the above, this research is an excellent alternative to the wide variety of previously studied techniques.

Future work will seek to include more complex algorithms and thus achieve a more robust technique and add simulations to verify its performance. In addition, future works will also seek to optimize the membership functions for the derivative of the error so that each individual will be made up of the genes corresponding to the error and its derivative. Finally, for future forecasts, it is expected that the controller, in its fuzzy part, includes a variable that corresponds to the weight added to the experimentation platform.

## Figures and Tables

**Table 1:** Linguistic expressions for the output, error and the derived of error.

Label	Linguistic Expression
vne	Very negative error
ne	Negative error
ezero	Zero error
pe	Positive error
vpe	Very positive error
vnde	Very negative derived of error
nde	Negative derived of error
dezero	Zero derived of error
pde	Positive derived of error
vde	Very positive derived of error
vno	Very negative output
no	Negative output
ozero	Zero output
po	Positive output
vo	Very positive output

**Table 2:** Fuzzy rules for the inference engine.

Error/Derror	vne	ne	ezero	pe	vpe
vpde	po	no	no	vno	vno
pde	po	ozero	no	vno	vno
dezero	vpo	po	ozero	no	vno
nde	vpo	vpo	po	ozero	no
vnde	vpo	vpo	po	po	no

**Table 3:** Comparison of controller performance with previous works.

Work	Rise Time (s)	Overshoot (%)	Settling Time (s)	Technique
Proposed work	0.19	15	0.63	Fuzzy logic and genetic algorithms
[45]	0.155	4.84	0.252	Fuzzy logic and genetic algorithms
[46]	0.21	15	0.64	Fuzzy logic and PSO
[47]	0.4	12.1	0.62	PID and genetic algorithms
[48]	0.418	17.4	3.17	PID and PSO

## Data Availability

The data presented in this research are available on request from the corresponding author.

## Abbreviations

The following abbreviations are used in this manuscript:

PID	Proportional-integral-derivative
IoT	Internet of things
MIMO	Multiple-input multiple-output
ANN	Artificial neural network
ACO	Ant colony optimization
COA	Center of area
SVR	Support vector regression
PMX	Partially mapped crossover
DAQ	Data acquisition
PWM	Pulse wide modulation
PPR	Pulses per revolution
DSC	Digital signal controller

## Appendix A

**Table A1:** Variables used in the control loop of the project.

Symbol	Meaning
*r*	Desired position
*y*	Actual position
*e*	Error
$\dot{e}$	Derivative of the error
$u_{c}$	Control signal delivered by the main control element
$u_{pwm}$	Control signal delivered by the DAQ
$u_{P}$	Control signal increased by the power stage
θ	Angular position

## Appendix B

The flow chart in Figure A1 shows how each variable used in this project interacts with the other. Color classification is added to differentiate the actions of each element described in the methodology.

## References

[1] Abushawish A., Hamadeh M., Nassif A. (2020). PID Controller Gains Tuning Using Metaheuristic Optimization Methods: A survey. Int. J. Comput..

[2] Levine W.S. (1996). PID Control, The Control Handbook.

[3] Zhang Y., Huang Y., Wang Y. (2022). Research on Compound PID Control Strategy Based on Input Feedforward and Dynamic Compensation Applied in Noncircular Turning. Micromachines.

[4] Ángeles Hurtado L., Rodríguez-Reséndiz J., Salazar-Colores S., Torres-Salinas H., Sevilla-Camacho P.Y. (2021). Viable Disposal of Post-Consumer Polymers in Mexico: A Review. Front. Environ. Sci..

[5] Rodriguez-Abreo O., Rodriguez-Resendiz J., Fuentes-Silva C., Hernandez-Alvarado R., Falcon M. (2021). Self-Tuning Neural Network PID with Dynamic Response Control. IEEE Access.

[6] Goud H., Sharma P., Nisar K., Ibrahim A., Haque M., Yadav N., Swarnkar P., Gupta M., Chand L. (2022). PSO Based Multi-Objective Approach for Controlling PID Controller. Comput. Mater. Contin..

[7] Latah M., Toker L. (2018). Artificial Intelligence Enabled Software Defined Networking: A Comprehensive Overview. IET Netw..

[8] Tadic V., Odry A., Burkus E., Kecskes I., Kiraly Z., Klincsik M., Sari Z., Vizvari Z., Toth A., Odry P. (2021). Painting Path Planning for a Painting Robot with a RealSense Depth Sensor. Appl. Sci..

[9] Lu Q., Sun Z., Zhang J., Zhang J., Zheng J., Qian F. (2022). A Novel Remote-Controlled Vascular Interventional Robotic System Based on Hollow Ultrasonic Motor. Micromachines.

[10] Chegini H., Mahanti A. (2019). A Framework of Automation on Context-Aware Internet of Things (IoT) Systems. Proceedings of the 12th IEEE/ACM International Conference on Utility and Cloud Computing Companion.

[11] Chegini H., Naha R.K., Mahanti A., Thulasiraman P. (2021). Process Automation in an IoT–Fog–Cloud Ecosystem: A Survey and Taxonomy. IoT.

[12] Liu C., Yue X., Zhang J., Shi K. (2021). Active Disturbance Rejection Control for Delayed Electromagnetic Docking of Spacecraft in Elliptical Orbits. IEEE Trans. Aerosp. Electron. Syst..

[13] Liu C., Yue X., Yang Z. (2021). Are nonfragile controllers always better than fragile controllers in attitude control performance of post-capture flexible spacecraft?. Aerosp. Sci. Technol..

[14] Baier-Fuentes H., Cascón Katchadourian J., Ángeles Martínez M., Herrera-Viedma E., Merigo J.M. (2018). A Bibliometric Overview of the International Journal of Interactive Multimedia and Artificial Intelligence. Int. J. Interact. Multimed. Artif. Intell..

[15] Odry A., Tadic V.L., Odry P. (2021). A Stochastic Logic-Based Fuzzy Logic Controller: First Experimental Results of a Novel Architecture. IEEE Access.

[16] Rodriguez-Abreo O., Hernandez-Paredes J., Rangel A., Fuentes-Silva C., Velasquez F. (2021). Parameter Identification of Motors by Cuckoo Search Using Steady-State Relations. IEEE Access.

[17] Mahapatra S., Daniel R., Dey D., Nayak S. (2015). Induction motor control using PSO-ANFIS. Procedia Comput. Sci..

[18] Yang C., Wang Y., Fan W. (2022). Long Stroke Design of Piezoelectric Walking Actuator for Wafer Probe Station. Micromachines.

[19] Shi K., Liu C., Sun Z., Yue X. (2022). Coupled orbit-attitude dynamics and trajectory tracking control for spacecraft electromagnetic docking. Appl. Math. Model..

[20] Chegini H., Beltran F., Mahanti A. Fuzzy Logic Based Pasture Assessment Using Weed and Bare Patch Detection. Proceedings of the International Conference on Smart and Sustainable Agriculture.

[21] Ma X.J., Sun Z.Q., He Y.Y. (1998). Analysis and Design of Fuzzy Controller and Fuzzy Observer. IEEE Trans. Fuzzy Syst..

[22] Vesely V., Ilka A. (2013). Gain-scheduled PID controller design. J. Process Control..

[23] Jain D.K., Mahanti A., Shamsolmoali P., Manikandan R. (2020). Deep Neural Learning Techniques with Long Short-Term Memory for Gesture Recognition. Neural Comput. Appl..

[24] Neenu T., Poongodi P. Position Control of DC Motor Using Genetic Algorithm Based PID Controller. Proceedings of the World Congress on Engineering.

[25] Park Y.M., Choi M., Lee K. (1996). An optimal tracking neuro-controller for nonlinear dynamic systems. IEEE Trans. Neural Netw..

[26] Panda S., Padhy N. (2008). Comparison of particle swarm optimization and genetic algorithm for FACTS-based controller design. Appl. Soft Comput..

[27] Rodríguez-Abreo O., Rodríguez-Reséndiz J., Montoya-Santiyanes L., Álvarez Alvarado J. (2022). Non-linear regression models with vibration amplitude optimization algorithms in a microturbine. Sensors.

[28] Masoudi S., Soltanpour M., Abdollahi H. (2018). A new adaptive fuzzy control method for a linear switched reluctance motor. IET Electr. Power Appl..

[29] Sierra-García J., Santos Peñas M. (2020). Switched learning adaptive neuro-control strategy. Neurocomputing.

[30] Tabrez M., Sadhu P.K., Hossain Lipu M.S., Iqbal A., Husain M.A., Ansari S. (2022). Power Conversion Techniques Using Multi-Phase Transformer: Configurations, Applications, Issues and Recommendations. Machines.

[31] Odeh S., Mora A., Moreno García M., Merelo Guervós J. (2015). A Hybrid Fuzzy Genetic Algorithm for an Adaptive Traffic Signal System. Adv. Fuzzy Syst..

[32] Rath A., Parhi D., Das H., Kumar P., Muni M., Salony K. (2019). Path optimization for navigation of a humanoid robot using hybridized fuzzy-genetic algorithm. Int. J. Intell. Unmanned Syst..

[33] Ponticelli G.S., Guarino S., Tagliaferri V., Giannini O. (2019). An optimized fuzzy-genetic algorithm for metal foam manufacturing process control. Int. J. Adv. Manuf. Technol..

[34] Zadeh L. (1965). Fuzzy sets. Inf. Control..

[35] Chen K., Huang C., He J. (2016). Fault detection, classification and location for transmission lines and distribution systems: A review on the methods. High Voltage.

[36] Mejía Ramírez C.A., Montes Rivera M., Medina Ramírez R.R., Ramírez Prado R.M., Gaitán Mercado C.M., Ochoa-Zezzatti A. (2021). Medicine Inventory Control System Through Fuzzy Logic and Genetic Algorithms: Applied to a Biopharmaceutical. Technological and Industrial Applications Associated with Intelligent Logistics.

[37] Dhimish M., Holmes V., Mehrdadi B., Dales M. (2018). Comparing Mamdani Sugeno fuzzy logic and RBF ANN network for PV fault detection. Renew. Energy.

[38] Praharaj M., Mohan B.M. (2021). Modeling and Analysis of Mamdani Two-Term Controllers Using Non-Uniformly Distributed Multiple Fuzzy Sets and CoA/CoG Defuzzification. IETE Tech. Rev..

[39] Liang H., Zou J., Zuo K., Khan M.J. (2020). An improved genetic algorithm optimization fuzzy controller applied to the wellhead back pressure control system. Mech. Syst. Signal Process..

[40] Mirjalili S.Z., Mirjalili S., Saremi S., Faris H., Aljarah I. (2018). Grasshopper optimization algorithm for multi-objective optimization problems. Appl. Intell..

[41] Zhang T., Liu Y., Rao Y., Li X., Zhao Q. (2020). Optimal design of building environment with hybrid genetic algorithm, artificial neural network, multivariate regression analysis and fuzzy logic controller. Build. Environ..

[42] Chin C.S., Lin W.P. (2018). Robust Genetic Algorithm and Fuzzy Inference Mechanism Embedded in a Sliding-Mode Controller for an Uncertain Underwater Robot. IEEE/ASME Trans. Mechatronics.

[43] Muhammad Zahir A.A., Alhady S., Othman W., Ahmad M.F. (2018). Genetic Algorithm Optimization of PID Controller for Brushed DC Motor. The Oxford Handbook of Innovation.

[44] Torres-Salinas H., Rodríguez-Reséndiz J., Estévez-Bén A.A., Cruz Pérez M.A., Sevilla-Camacho P.Y., Perez-Soto G.I. (2020). A Hands-On Laboratory for Intelligent Control Courses. Appl. Sci..

[45] Sheroz K., Salami A., Adetunji L., Alam A., Salami M., Hameed S., Hasan A., Islam M. (2008). Design and Implementation of an Optimal Fuzzy Logic Controller Using Genetic Algorithm. J. Comput. Sci..

[46] Bouallègue S., Haggege J., Ayadi M., Benrejeb M. (2012). PID-type fuzzy logic controller tuning based on particle swarm optimization. Eng. Appl. Artif. Intell..

[47] Peng J., Dubay R. (2011). Identification and adaptive neural network control of a DC motor system with dead-zone characteristics. ISA Trans..

[48] Joseph S., Mishra M., Omizegba E. (2011). Automatic Tuning of Proportional-Integral-Derivative (PID) Controller Using Particle Swarm Optimization (PSO) Algorithm. Int. J. Artif. Intell. Appl..

